# JOINclusion: A serious mobile game for promoting ethnocultural empathy in schools

**DOI:** 10.1111/bjdp.70055

**Published:** 2026-05-31

**Authors:** Alessandra Colella, Concetta Esposito, Annaleda Mazzucato, Ioannis Christidis, Enrique Hortal, Helen C. Leligou, Davide Marocco, Michela Ponticorvo, Yusuf Can Semerci, Dario Bacchini

**Affiliations:** ^1^ Department of Humanities University of Naples “Federico II” Naples Italy; ^2^ Fondazione Mondo Digitale Rome Italy; ^3^ Department of Industrial Design and Production Engineering University of West Attica Athens Greece; ^4^ Department of Advanced Computing Sciences Maastricht University Maastricht Netherlands

**Keywords:** ethnocultural empathy, gamified education, inclusive education, multicultural, serious games, social emotional learning (SEL)

## Abstract

This study examines the efficacy of JOINclusion, a serious mobile game designed to enhance ethnocultural empathy in children aged 8 to 12. Grounded in the Social Emotional Learning (SEL) framework, JOINclusion delivers interactive scenarios through a narrative‐driven Story Mode and a collaborative Multiplayer Mode, encouraging emotional recognition, cognitive perspective‐taking and prosocial decision‐making. The game was evaluated using a pre–post design with 286 children across three European countries (Italy, Greece and the Netherlands). Two key outcomes were assessed: children's perceived educational benefit from the game and changes in ethnocultural empathy, specifically in empathic feeling/expression and perspective‐taking. Findings indicated that most children reported perceived improvements in empathy‐related skills after gameplay. Results from the pre‐ and post‐comparison using Latent Change Score Models showed a significant increase in empathic perspective‐taking and a marginal improvement in empathic feeling and expression. These effects were significant regardless of gender, age and migratory background. These findings support the potential of gamified learning experiences to foster inclusive social behaviour by promoting empathy‐related competences. Despite its limitations, JOINclusion offers a promising, scalable model for integrating empathy training into diverse educational settings through engaging and child‐centred technology.

**Statement of Contribution**

**What is already known on this subject**

1. Empathy supports prosocial behaviour and reduces intergroup prejudice in children.
2. Middle childhood is a sensitive period for empathy development and prejudice formation.
3. Game‐based interventions can effectively promote social–emotional learning and empathy development.

**What the present study adds**

1. Empirical evaluation of a serious game specifically targeting ethnocultural empathy in children.
2. Demonstrates the feasibility of integrating SEL frameworks with ethnocultural empathy via digital gaming.
3. Cognitive empathy shows greater responsiveness to game‐based training than affective empathy.

## INTRODUCTION

Europe is witnessing a growing diversity in its ethnic and cultural population. According to the most recent data, nearly 87 million international migrants resided in Europe in 2020—an increase of nearly 16% since 2015. Of these, around 44 million were born in Europe but living elsewhere within the region, up from 38 million in 2015. The number of non‐European migrants living in Europe also grew, reaching 40 million in 2020 (McAuliffe & Oucho, 2024). In response to this demographic change, the European Commission (2020) has positioned equality and inclusion as key priorities in the European agenda, recognizing the need to create pathways for young migrants to fully participate in European society and realize their potential as contributing members of diverse communities.

In this context, it is crucial for education systems to equip students with the social and emotional competencies for effectively navigating multicultural environments. Among these competences, empathy represents a core skill that supports prosocial interactions and inclusive attitudes (Bennett, 2016; World Health Organization, 1993). Digital serious games, which combine narrative engagement and learner agency with immediate feedback on each choice, offer promising tools for fostering such competences in engaging scalable formats (Connolly et al., 2012).

This study explores the efficacy of JOINclusion, a serious mobile game designed to enhance ethnocultural empathy in children aged 8–12. Existing game‐based empathy interventions have rarely targeted ethnocultural empathy specifically or systematically integrated established social–emotional learning (SEL; CASEL, 2020) frameworks. JOINclusion fills these gaps by combining SEL principles with developmentally informed game design. It specifically offers three theoretical contributions: (1) operationalizing ethnocultural empathy into scenario‐based learning experiences that simulate intercultural classroom dilemmas; (2) aligning game mechanics (narrative branching, feedback contingent on each player choice and collaborative problem‐solving) with developmental theories of empathy (Eisenberg, 2006; Hoffman, 2000), perspective‐taking (Selman, 1980) and constructivist models of moral reflection grounded in peer interaction and guided justification (Kohlberg, 1984; Piaget, 1932); and (3) extending SEL implementation from teacher‐led curricula into a scalable technology‐mediated format.

The target age range represents a critical period for developing social competence and empathy‐related abilities (Denham et al., 2007; Eisenberg et al., 2014), as well as a sensitive window for the emergence of ethnic‐based prejudice (Raabe & Beelmann, 2011). The aim of JOINclusion is to cultivate children's capacity to emotionally connect with and understand peers from diverse backgrounds, thereby fostering more inclusive interactions in culturally diverse school environments.

### Children's experiences of out‐group others

To better understand the development of discriminatory attitudes and ethnic prejudice in children and adolescents, Nesdale (2004) proposed the Social Identity Development Theory (SIDT), expanding the classical Social Identity Theory (Tajfel, 1978). According to this theory, children's ethnic prejudice develops through four stages: From birth to around age two, children have little awareness or meaning assigned to social groups (Stage 1). At around age three, children begin to differentiate others based on objective indicators of social groups (e.g., skin colour; Stage 2). Next, children begin to identify with their own social group (and show a preference for their group), occurring around age four or five (Stage 3). By around age seven, children's cognitive development reaches a stage where biases could crystallize, making them capable of possessing and expressing prejudice against out‐groups (Stage 4). Therefore, according to the SIDT, this is the developmental period when discriminatory biases and prejudice are most likely to emerge if environmental or social factors are present.

These considerations imply that, especially from age seven, children's empathy‐related responding might differ based on the characteristics of the targets (Eisenberg et al., 2015; Peplak & Malti, 2022), with children being more likely to show liking, positive thoughts and perspective‐taking (i.e., understanding another's thoughts and emotions) towards members of their same perceived race or ethnicity. However, to date, only a few studies have focused on children's differential empathy based on race or ethnicity (O'Driscoll et al., 2021). In their review of empathy interventions with children, Aboud and Spears Brown (2013) noted that interventions that focus on increasing children's empathy, particularly if they offer specific or concrete examples of discrimination and exclusion, are most effective at reducing bias in young children compared to other interventions. Also, it has been found that individuals who can feel sympathy or anger regarding social injustices are more likely to take action against them (Bañales et al., 2021). Based on these literature findings, it is reasonable to assume that children who have the opportunity to develop an emotional connection with marginalized groups and who are motivated by concerns about equity and fairness are likely to continue to experience concern for victims and awareness of discriminatory biases (Abrams et al., 2015; Hazelbaker et al., 2022).

In this sense, schools certainly provide knowledge about diversity and stimulate student motivation and effort (Nishina et al., 2019). In highly diverse schools, learning about different cultures takes place informally at all times, but this process can also be encouraged through classroom discussions, cultural activities and planned exposure to different identities and teaching strategies. According to Solhaug and Osler (2018), students' ability to understand and appreciate cultural diversity is a significant predictor of their intercultural empathy, creating opportunities for schools to promote social inclusion.

### Promoting ethnocultural empathy

Most psychological interventions designed to improve interethnic relationships among young people have focused primarily on reducing prejudice and discrimination (Aboud & Spears Brown, 2013). Although such approaches are essential for promoting a more equitable and respectful school climate, they may be insufficient (Nishina et al., 2019). Beyond specific diversity or anti‐bias training, a general focus on social competence and prosocial behaviour, such as perspective‐taking, social problem‐solving and cooperative learning, has been shown to be effective in fostering social inclusion in ethnically diverse educational settings (Nishina et al., 2019). These skills provide students with the tools to respond sensitively to others, building the foundation for inclusive social interactions. Additionally, such approaches may be especially appropriate for younger children, who are still developing an adult‐like understanding of ethnic differences (Quintana, 1998) and may benefit more from generalized social–emotional learning rather than identity‐specific interventions.

According to Barrett (2016, 2018), intercultural competence includes a set of values, attitudes, skills and knowledge needed to understand culturally different others and engage with them respectfully and constructively. Among these, empathy plays a central role. Notably, Barrett (2018) noted that empathy can be nurtured from a young age in preschool and primary education, while knowledge and critical understanding of culture develop later and are more appropriately addressed in upper secondary education or even higher education (Barrett, 2018).

Among the skills Barrett identified, empathy plays a central role. Empathy is broadly defined as the capacity to recognize and share others' emotional states or perspectives, typically involving both cognitive and affective components (Hoffman, 2000). The cognitive aspect refers to the ability to adopt another person's perspective or infer their mental state, whereas the affective component involves experiencing an emotional resonance with the other's feelings (Eisenberg, 2006).

Although empathy is associated with a range of prosocial outcomes, including reduced prejudice (Wang et al., 2003), increased helping behaviour (Litvack‐Miller et al., 1997) and more inclusive attitudes towards members of ethnic out‐groups (Stephan & Finlay, 1999), it is not intrinsically inclusive. Individuals tend to empathize more with those who are socially or culturally similar to themselves—a phenomenon known as empathic bias (Hoffman, 2000). This bias can perpetuate in‐group favouritism and undermine efforts to build genuine intergroup understanding, particularly in multicultural settings. To overcome this limitation, researchers have pointed out the importance of perspective‐taking and other cognitive strategies that help extend empathy‐related responding beyond in‐group boundaries (e.g., Miklikowska, 2018), giving rise to the construct of ethnocultural empathy—also referred to as cultural competence, cultural empathy or trans‐cultural empathy (Dyche & Zayas, 2001).

Ridley and Lingle (1996) were the first to use and define the concept of cultural empathy, arguing that cultural empathy goes beyond general empathy and includes understanding and acceptance of another person's cultural point of view. Wang et al. (2003) coined the term ‘ethnocultural empathy’ from theories on general and cultural empathy, defining it as empathy directed towards people from ethnic cultural groups different from one's ethnocultural group. Their studies' findings culminated in the identification of four key components of ethnocultural empathy: Empathic Feeling and Expression (EFE), focusing on the expression of ethnocultural empathic thoughts and feelings towards members of other ethnic groups; Empathic Perspective Taking (EPT), which is the ability to understand how a person with a different ethnic background thinks or feels; Acceptance of Cultural Differences (ACD), referring to concern with accepting why people from other ethnic groups behave as they do, for example, wearing traditional clothing or speaking their own language; Empathic Awareness (EA), which reflects being conscious of how society, media and the job market treat other ethnic groups.

Developing this form of empathy is crucial for preparing young people to interact respectfully and constructively in multicultural societies. JOINclusion is a serious mobile game specifically designed with this goal in mind, developed as part of an Erasmus+ project that reflects the European Commission's commitment to promoting inclusion and diversity. Specifically, the JOINclusion serious game is grounded in the Social Emotional Learning (SEL; CASEL, 2020) framework, which describes five interrelated competences across emotional, behavioural and cognitive domains, with social awareness– including empathic engagement with culturally diverse others– directly relevant to the present work.

### Digital games as tools for empathy development

Over the past decade, scholarly interest has increased in exploring how games and digital interventions can support psychological well‐being, emotional competence, and more generally, prosocial behaviour (Carissoli & Villani, 2019; Harrington & O'Connell, 2016; Villani et al., 2018). Serious games are commonly considered promising formats, capable of stimulating both emotional and cognitive growth. Especially for children and adolescents, interactive digital environments serve as highly engaging platforms that promote prosocial skills through experiential and immersive learning.

This trend is consistent with the Positive Technology Movement, which posits that digital tools can actively enhance psychological well‐being and personal growth (Gaggioli et al., 2019). Positive technology integrates game‐based pedagogies, virtual simulations and interactive content to produce educational interventions with emotional depth. These tools are explicitly designed to train emotional intelligence and empathy by guiding users through socially rich experiences that nurture personal and relational growth.

Although concerns have been raised about digital media's effects on emotional recognition (Turkle, 2015; Uhls et al., 2014), well‐designed interactive experiences can foster empathic engagement that transfers to face‐to‐face interactions (Carrier et al., 2015).

Games, in particular, have been recognized as immersive formats conducive to empathy‐building. According to Belman and Flanagan (2010), digital gameplay allows users to adopt alternative viewpoints and embody emotionally complex roles. This immersive quality enhances emotional resonance and moral engagement. Supporting this view, Bachen et al. (2012) found that high school students who participated in simulation‐based learning activities reported increased global empathy and greater interest in multicultural understanding and global issues. Taken together, findings from this body of research indicate that although technology‐mediated communication carries potential risks, well‐designed digital interventions—especially those grounded in serious games and interactive storytelling—can support the development of empathy and prosocial behaviour.

### JOINclusion: Development, design and technical foundation

JOINclusion is a 2D mobile application developed using the Unity game engine and optimized for the Android operating system. Drawing inspiration from visual novel games, it emphasizes interactive storytelling to engage players emotionally and cognitively. The game currently supports four languages: English, Dutch, Greek and Italian.

The game scenarios and narrative architecture were collaboratively designed by a multidisciplinary team including educational psychologists and developmental researchers, working with game designers and elementary school educators from Greece, Italy and the Netherlands. The development process involved three phases: (1) literature review and theoretical mapping, in which the research team identified key empathy competences from developmental literature (Eisenberg, 2006; Hoffman, 2000); (2) co‐design workshops with teachers and educators (*N* = 15 across the three countries), who provided input on age‐appropriate language, culturally relevant contexts and common classroom social challenges; and (3) pilot testing with students from primary schools (*N* = 30 across the three countries; *M* age = 10.09, SD = 1.29). Pilot testing evaluated enjoyment, willingness to replay, curiosity, immersion, perceived skill development and real‐world relevance through self‐report questionnaires. Technical functionality, user interface intuitiveness and scenario age‐appropriateness were assessed through brief teacher interviews, leading to refinements in visual design (e.g., larger text, simplified navigation icons) and scenario wording (e.g., reduced vocabulary complexity).

The game adopts real‐life school scenarios to create an immersive educational experience. The simulation incorporates multimodal affective cues—including auditory stimuli and dynamic facial expressions representing different emotional states—to support accurate emotional recognition and appropriate responsive behaviours. The interactive architecture includes four agent types: (1) a user‐controlled protagonist; (2) a focal character representing ethnocultural diversity who is the primary recipient of empathic responses; (3) peripheral non‐player characters (NPCs) providing social context and reinforcement; and (4) a narrator who guides the learning process.

At key decision points, players choose among multiple response options, each leading to different narrative paths and character reactions. These choices operate within a semi‐adaptive feedback system—responsive to specific errors but not dynamically personalized—using a decision‐tree architecture that provides contingent, scripted responses based on player choices. When players misidentify emotions or fail to intervene prosocially, the system triggers pre‐programmed narrator feedback (e.g., perspective‐taking prompts, emotion recognition guidance) targeting the specific competency gap demonstrated. Teachers receive training to guide post‐gameplay reflection and discussion, but do not intervene during active gameplay, preserving the designed learning experience.

### The game's architecture and learning goals

The JOINclusion serious mobile game is implemented through a dual‐modal interactive platform consisting of a Story Mode and a Multiplayer Mode. Both modalities are designed to promote ethnocultural empathy and prosocial behaviour, grounded in social cognitive and socioemotional developmental theories that inform effective empathy training during middle childhood (see Figure 1). The Story Mode component serves as a training mechanism through which participants acquire and refine their competencies; the Multiplayer Mode is an applied cooperative learning environment in which students engage in synchronous collaborative activities that require the practical implementation of previously acquired empathic skills.

**FIGURE 1:**
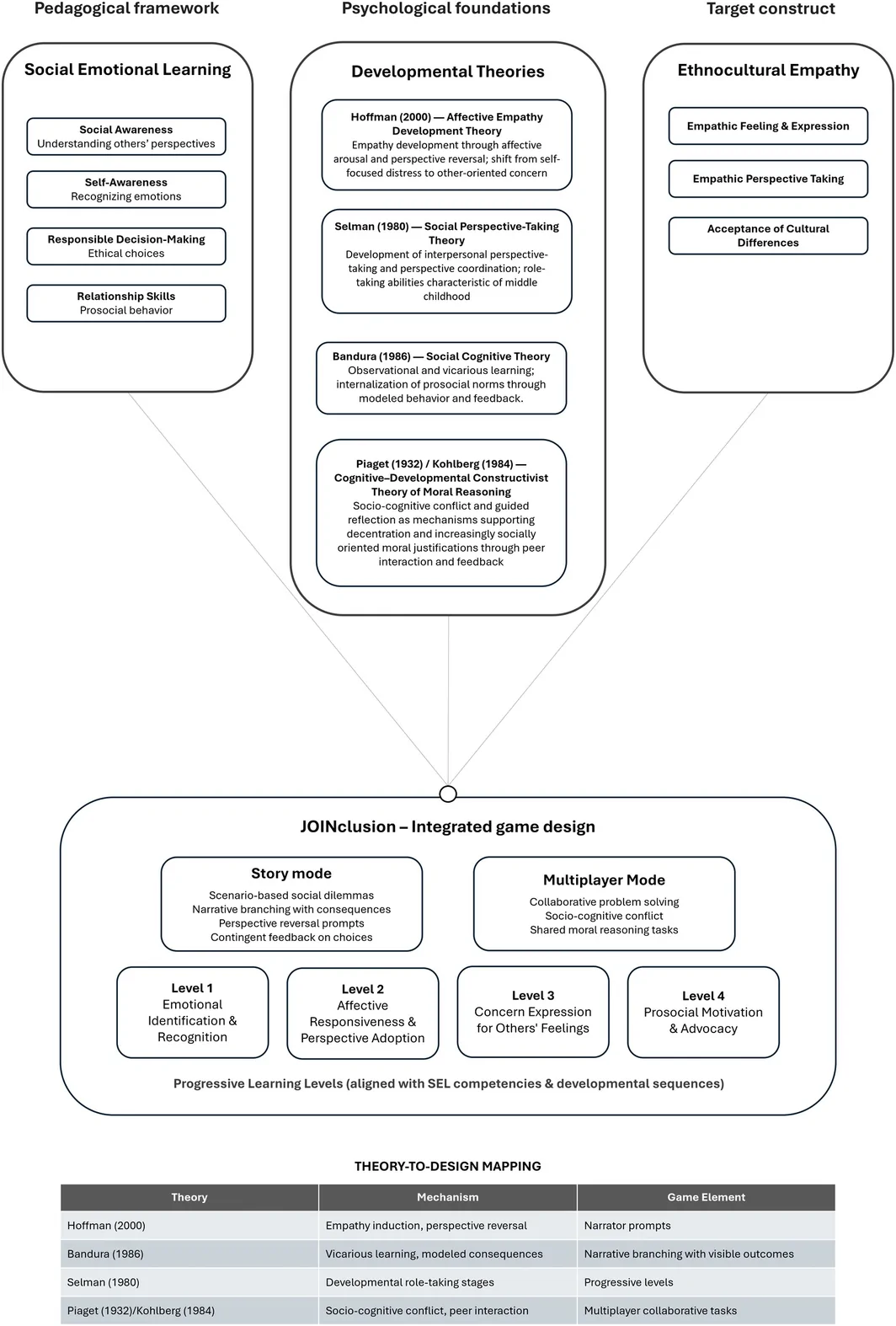
Theoretical foundations and design logic of the JOINclusion digital game. The figure depicts how three conceptual streams—Social and Emotional Learning (SEL) competences (only the most relevant competences are shown), developmental and socio‐cognitive psychological theories and ethnocultural empathy as the target construct—informed the design of JOINclusion. SEL competences specify educational goals, psychological theories explain the developmental mechanisms underlying the game, and ethnocultural empathy defines the primary outcomes. These elements are translated into story‐based and multiplayer game modes and into progressive learning levels aligned with developmental abilities.

Story Mode presents children with interactive scenarios that simulate typical classroom and social situations where empathy and prosocial behaviour are challenged. The narrative architecture is structured across four progressive levels, each designed to cultivate increasingly complex empathic competences aligned with the SEL framework (CASEL, 2020).

Level 1—Emotional Identification and Recognition—facilitates the identification of affective states and the recognition of multimodal expressive cues (verbal, paralinguistic and physiological cues) as well as contextual social determinants that indicate others' emotional experiences. Level 2—Affective Responsiveness and Others' Perspective Adoption—fosters both self‐oriented and other‐oriented emotional responses while developing cognitive perspective‐taking capabilities across ethnocultural boundaries. Specifically, this level addresses the critical developmental achievement of distinguishing between self‐oriented empathic distress and other‐oriented empathic concern (Eisenberg, 2006), a differentiation that emerges around ages 7–9 and predicts prosocial behaviour (Hoffman, 2000). The explicit focus on ethnocultural perspective‐taking recognizes that while basic perspective‐taking consolidates by middle childhood (Selman, 1980), applying these skills across cultural boundaries requires specific guidance and practice (Quintana, 1998). Level 3—Concern Expression for the Feelings of Others—focuses on demonstrating empathic understanding and compassionate concern towards individuals from diverse ethnocultural backgrounds. The learners practice expressing empathic responses that acknowledge cultural differences in emotional experience and expression. This level operationalizes the transition from cognitive understanding to affective concern, which Hoffman (2000) identifies as central to mature empathic functioning, while also developing the communicative competence emphasized in SEL frameworks (CASEL, 2020). Level 4—Prosocial Motivation—develops intrinsic motivation towards enhancing collective well‐being, handling conflicts constructively, resisting negative social influences and advocating for the equitable treatment of culturally diverse others.

In the first scenario, the target character is deliberately excluded by peers and subsequently harassed by a classmate, who intentionally drops the target character's books onto the floor. The player is then presented with a choice of actions that will determine the outcome: If the player actively intervenes (e.g., requesting that the aggressors stop and show respect towards the victim), the target character responds with a smile and the aggressors ultimately cease their actions and offer an apology, resulting in a positive ending; if the player fails to intervene or chooses to join in the mockery, the target character experiences heightened distress, bursts into tears and exits the class, leading to a negative ending; if the player exhibits a minimal empathic response but refrains from taking explicit action against the bullying behaviour, the target character will display only a faint smile, indicative of partial but insufficient emotional support. This narrative structure draws on Bandura's (1986) concept of vicarious learning by allowing players to observe the consequences of different behavioural choices without experiencing real‐world social risks. The second scenario revolves around a group project, in which a foreign student is asked to read aloud in front of the class. Due to language difficulties, the target character struggles with pronunciation, leading to heightened anxiety and embarrassment. Other classmates mock the target character's accent, despite the teacher's attempts to maintain order. Once again, the player's decision to either intervene or remain passive dictates the course of the interaction: If the player joins in the mockery or remains indifferent, the target character fails to complete the reading, becomes overwhelmed with distress and exits the classroom in tears, leading to a negative outcome; if the player confronts the classmates, urging them to cease their disruptive behaviour and empathize with the target character, the aggressors ultimately desist and offer an apology, leading to a positive resolution; if the player merely asks the classmates to stop disrupting the lesson, the target character continues reading but exhibits a sad expression, indicating residual emotional distress.

Both scenarios incorporate a feedback system designed to facilitate perspective‐taking and emotional recognition. In cases where the player neglects to support the target character or engages in exclusionary behaviour, the narrator's character prompts reflection by asking the player to consider how they would feel if they were subjected to a similar experience. This ‘perspective reversal’ technique directly applies Hoffman's (2000) empathy induction method, which research demonstrates is particularly effective for children ages 8–12 because they have achieved sufficient role‐taking ability (Selman's Stage 2–3) to mentally simulate others' experiences but still benefit from explicit prompts to do so. The narrator further encourages the player to acknowledge the importance of social support, suggesting that in similar situations, they may also benefit from the presence of a supportive figure who can provide reassurance or advocate on their behalf when necessary.

Additional guidance is provided if the player fails to identify the target character's emotional state correctly. Specifically, when the target character exhibits sadness, but the player misattributes the emotion, the narrator systematically explains the physical cues associated with sadness (e.g., lowered gaze, the corners of the mouth turned down and a slumped posture). Additionally, the narrator contextualizes the experience, indicating that the target character may feel anger towards the aggressors as a secondary emotional response to exclusion.

When players reinforce bullying behaviour, the narrator highlights the lack of self‐awareness exhibited by the aggressors, emphasizing that their actions fail to account for the emotional consequences imposed on the target character. The narrator further prompts the player to consider how they might react in similar circumstances, reinforcing the importance of prosocial intervention and moral responsibility. This feedback mechanism is informed by Kohlberg's (1984) account of moral reasoning development, in that it invites children to move beyond immediate self‐focused considerations towards greater attention to the interpersonal and social consequences of their actions. By emphasizing the impact of teasing on others and referencing shared expectations about respectful behaviour, the narrator supports more socially attuned moral reasoning within developmentally appropriate contexts. This approach is consistent with work showing that middle childhood is a sensitive period for moral‐cognitive development, particularly when children engage in structured moral dilemmas and perspective‐taking exercises (Gibbs et al., 2007). Multiplayer Mode is designed to reinforce and extend the individual social–emotional skills acquired in Story Mode by engaging students in collaborative problem‐solving tasks. In this mode, children are organized into groups (up to 10 groups simultaneously, with a maximum of 3 participants per group) and work together to address social dilemmas—such as bullying or exclusion—presented through interactive scenarios. The core mission for students is to save the school from a witch by collaboratively resolving such social dilemmas. To do so, they must find and tap on gems scattered throughout the virtual school environment. Each gem activates a shared scenario that all participants must analyse and respond to through a related question. If most students answer correctly, the scenario is marked as successfully resolved; otherwise, it is considered unsuccessful. The collaborative structure reflects Piaget's (1932) theory that peer interaction promotes moral development by exposing children to conflicting viewpoints, necessitating negotiation and perspective coordination—processes that facilitate the transition from heteronomous to autonomous moral reasoning (Killen & Smetana, 2015). The requirement for group consensus on empathy‐related judgements creates ‘socio‐cognitive conflict,’ that stimulates cognitive‐moral growth during middle childhood (Kruger, 1992).

The number of scenarios corresponds to the number of gems placed within the game. Educators can configure this during game setup, with a maximum of five gems allowed per session to ensure appropriate game duration. To win, students must locate all gems and collectively answer all associated questions correctly. Failure to meet these objectives results in an unsuccessful game outcome. Additionally, educators can tailor the Multiplayer Mode experience by selecting social dilemmas from a dedicated repository, based on the sociocultural dynamics and what they consider more relevant in their classroom context. These scenarios can then be integrated into the game and accessed by students, thus enhancing both the educational impact and contextual relevance of the experience. Comprehensive details regarding the JOINclusion game—including its interface, narrative structure and sample scenarios—are available on the official project website (https://dke.maastrichtuniversity.nl/JOINclusion/).

### The present study

This study focused on two specific outcomes: (1) students' perceived educational value of the JOINclusion game, especially in relation to empathy‐related skills, and (2) changes in ethnocultural empathy levels following gameplay. For this second outcome, a pre‐post design was employed, which allows for a comparison of participants' ethnocultural empathy levels before and after their involvement in the game's pilot study. Additionally, demographic factors (i.e., gender, age and migratory background) were included as covariates to examine whether individual characteristics had any effect on the observed outcomes.

## METHOD

### Participants and procedure

Data collection, game piloting and efficacy testing were conducted as part of the JOINclusion Erasmus+ project, funded by the European Commission, which aimed to promote social inclusion in primary education through gamified learning experiences. Inclusion criteria for participation in the study required participants to be (1) enrolled in primary school within the target age range of 8–12 years, (2) able to understand and communicate in the language of instruction used in their respective countries and (3) able to engage with digital learning platforms as part of regular classroom activities. The study's sample consisted of 286 primary school children from Greece (*N* = 99; 34.6%), Italy (*N* = 142; 49.7%) and the Netherlands (*N* = 45; 15.7%) aged 8 to 12 years (M = 9.36, SD = 0.48). The distribution was equal between girls and boys (50.7% boys, 49.3% girls). Additionally, 32.1% of participants had a migratory background; specifically, approximately 20.9% of children with a migratory background were first‐generation immigrants (both the children and parents were foreign‐born). More details on sample demographics are provided in Table 1.

**TABLE 1:** Percentages of children with a migratory background (*N* = 286).

Children migratory background	%
No migratory background	67.9
Children native‐born, one parent foreign‐born	5.1
Children native‐born, both parents foreign‐born	5.8
Children foreign‐born, neither parent foreign‐born	‐
Children and one parent foreign‐born	0.4
Children and both parents foreign‐born	20.9
Children ethnicity	
European	62.9
North African, Middle Eastern and Central Asian	7.9
Sub‐Saharan African	4.5
South and South‐East Asian	9
East Asian	3.4
Latin American	9
North American and Australasian	3.4

The study protocol for this study was approved by the University Ethical Committee for Psychological Research at the University of Naples ‘Federico II’ (protocol number: 16/2023). To protect participants' privacy, a robust pseudonymization procedure was implemented in accordance with ethical standards for research involving minors (American Psychological Association, 2017; European General Data Protection Regulation, 2016/679).

The project team conducted preliminary meetings with participating schools to present the JOINclusion project, raise awareness about the study's objectives, and enhance understanding of the project's procedure and phases. Following institutional approval, informed consent was obtained from parents or legal guardians of the participants, and students provided their assent prior to participation.

The data collection process worked as follows: Initially, each student was assigned a unique nickname, generated by combining various animal names with colours, resulting in unique identifiers like ‘red_owl’ or ‘yellow_turtle’. This method ensured that they could be easily referenced in all subsequent research activities without revealing their true identities. As part of the study, teachers were asked to complete a demographic questionnaire for each student. This questionnaire collected essential information such as age, gender and ethnic background. By linking these demographic profiles with the students' responses to pre‐ and post‐test questionnaires, researchers were able to track changes in the expected outcomes over time while accounting for sociodemographic factors.

Data collection occurred during a two‐week period in April and May 2024. The experimentation followed a two‐phase protocol.

Week 1 (Day 1): In order to establish baseline measurements before gameplay, participants completed an online pre‐assessment questionnaire, which was administered on tablets. Because of the children's young age (8–12 years), teachers read all instructions aloud to the class before administration and were available to clarify individual questions about which items were being asked (without influencing responses). Research assistants circulated during completion to monitor for confusion and to offer procedural assistance if needed. Once the questionnaire was completed, children moved on to Scenario 1 of the game and, finally, to a collaborative experience in Multiplayer Mode, where they worked together to solve challenges.

Week 3 (Day 2): In this phase, children played Scenario 2 and participated in a second collaborative Multiplayer session. Once gameplay was finished, they completed a post‐assessment questionnaire, which was developed to evaluate both their subjective experience with the game and outcomes related to ethnocultural empathy as targeted by the game experience.

Game sessions were supervised by classroom teachers who received standardized training on the JOINclusion game protocol prior to implementation. Teacher training sessions were conducted by expert psychologists, and comprehensive training materials, including a free online course, were provided to ensure they could effectively incorporate the tool into their classrooms. All participating students were exposed to the same game scenarios in a fixed sequence, ensuring consistent content delivery across all three countries. To monitor implementation fidelity, we tracked completion rates for each scenario through the game's built‐in analytics system, which recorded time on task, scenario completion and student engagement indicators (e.g., number of choices made, progression through dialogue trees).

### Measures

The questionnaires were initially developed in English and translated into Dutch, Greek and Italian. The translation and cultural adaptation process involved bilingual members of the research team who were native speakers of the target languages. After the initial translations, teachers in each country reviewed the materials to ensure clarity, age appropriateness and cultural sensitivity before the questionnaires were finalized and administered.

#### Perceived educational scope of the game

At post‐test assessment, participants were asked to assess whether their experience with the JOINclusion game enhanced their empathy‐related abilities. This included their capacity to recognize and interpret others' emotions, appreciate diverse perspectives and cultivate greater motivation to support those around them. The assessment included three questions designed to gauge the game's impact on their empathetic skills: (i) To what extent do you believe that playing JOINclusion has helped you in recognizing and understanding the emotions of others? (ii) In your opinion, has JOINclusion helped you to appreciate different viewpoints and respond more empathetically? (iii) Following your experience with the game, do you feel a heightened motivation to contribute to the happiness and well‐being of others? These questions were developed specifically for this study as an exploratory measure of participants' subjective perceptions of the game's educational impact. Responses were collected using a 3‐point scale: 1 = No, 2 = Maybe and 3 = Yes.

#### Ethnocultural empathy

Ethnocultural empathy was evaluated both before and after the game experimentation using a child‐adapted version of the Italian Ethnocultural Empathy Scale (Albiero & Matricardi, 2013; original scale by Wang et al., 2003), specifically designed for adolescents and adults. The original questionnaire consisted of 31 items across four dimensions: EFE, EPT, ACD and EA. For this study, eight items distributed across three dimensions were selected: EFE (3 items: I feel very sorry when I see someone being treated badly because of their ethnicity; If I see someone laughing at someone else's origins, I ask them to stop, even if it doesn't directly affect me; I like to help people of a different ethnicity than mine if I think someone is fooling them), EPT (3 items: I can imagine how some people might feel angry that they don't have the same opportunities because of the colour of their skin or the country they're from; I can easily imagine how it feels to be a foreign child; It's easy for me to imagine myself in the life stories of people who come from countries other than mine) and ACD (2 items: It bothers me to talk to someone from a different country than mine, even if they speak my language well; I do not like that people from other countries or with different origins prefer their traditional clothes over those of my country). The EA dimension, which reflects being conscious of how society, media and the job market treat other ethnic groups, was excluded from this study due to its developmental inappropriateness for children aged 8–12 years. Research on children's media literacy indicates that although young audiences can be influenced by media representations of ethnic minorities (Durkin et al., 2012), their ability to critically analyse these representations and understand their broader societal implications develops gradually throughout childhood and adolescence (Dickinson et al., 2023).

Participants rated each item on a three‐point Likert scale, where 1 = ‘No’, 2 = ‘So‐so’ and 3 = ‘Yes’, indicating the extent to which each statement reflected their experiences. The criteria for item selection included relevance to the dimensions of interest, clarity and appropriateness for the age group. After selecting the items, a small pilot study was conducted with a convenience sample of eight children to identify any issues with comprehension or engagement. Feedback from parents and educators was also considered to refine the items further.

When the scale was administered to the larger JOINclusion sample, several children from all three countries involved showed inconsistent response patterns on the two items within the ACD dimension, which may have been due to the reverse wording of the items. As a result, these two items were excluded from the analyses in the current study.

The factorial structure of the scale at pre‐test showed acceptable fit, SB*χ* ^2^(8) = 20.741, *p* = .008, RMSEA = .08 (90% CI [0.04, 0.12]), CFI = .93 and SRMR = .05. More details are reported as Supplemental Material (Table S1). Measurement invariance across countries, tested using the alignment method, was supported. Only one significant difference emerged: Dutch children reported a higher intercept score than Greek children on Item 1 of the EFE scale (i.e., feeling sorry about ethnic mistreatment).

Cronbach's alphas for pre‐ and post‐test scales ranged between .62 and .67, suggesting adequate internal consistency.

### Attrition analysis

Attrition analysis was conducted to examine missing data patterns between pre‐test and post‐test assessments. While pre‐test completion rates were high, with only 3.5%–4.5% missing data across the ethnocultural empathy items, substantial attrition occurred at post‐test, with approximately 40% of participants not completing the follow‐up assessment. Little's Missing Completely at Random (MCAR) test was significant, *χ* ^2^ (76) = 128.960, *p* < .001, indicating that data were not missing completely at random. *t*‐tests revealed that older children were significantly more likely to miss the post‐test assessment (*p* < .001). Additionally, children who missed the post‐test had significantly lower pre‐test scores on Item 1 (i.e., *feeling sorry about ethnic mistreatment*) and Item 2 (i.e., *intervening against ethnic teasing*) of the EFE scale, but higher pre‐test scores on Item 3 of the EPT scale (i.e., *imagining others' life stories*) (all *p*s < .001). Given the non‐random nature of missing data, Full Information Maximum Likelihood (FIML) was employed rather than listwise deletion for subsequent analyses.

The observed attrition pattern can be attributed to a number of factors related to the constraints imposed by the school scheduling system. Examples of such factors include scheduling constraints that overlap curricular activities and unforeseen student absences.

### Analytic strategy

The analytical approach adopted in this study involved two main phases: (1) analysis of the perceived educational value of the JOINclusion game, and (2) evaluation of the efficacy of the gamified learning experience.

All descriptive analyses were conducted using IBM SPSS Statistics, version 29 (IBM Corp., 2023). Before assessing the efficacy of the JOINclusion game experience, the distributional properties of all variables were examined using Shapiro–Wilk tests. Descriptive statistics, including item‐level means and standard deviations, were computed to explore the preliminary characteristics of the study variables. This item‐level approach is consistent with the subsequent analytic strategy, which assessed the game's efficacy in fostering ethnocultural empathy through multiple‐indicator Latent Change Score (LCS; Figure 2) models (Steyer et al., 2000) conducted in Mplus version 8 (Muthén & Muthén, 1998–2021). For each dimension of ethnocultural empathy (EFE and EPT), we constructed a separate LCS model that directly parameterized change while accounting for measurement error, individual differences in baseline levels and measurement invariance across time points. Each model was specified with two latent factors: (1) a latent intercept factor (TAU) representing true baseline levels, and (2) a latent change factor (CHANGE) representing true change from pre‐test to post‐test.

**FIGURE 2:**
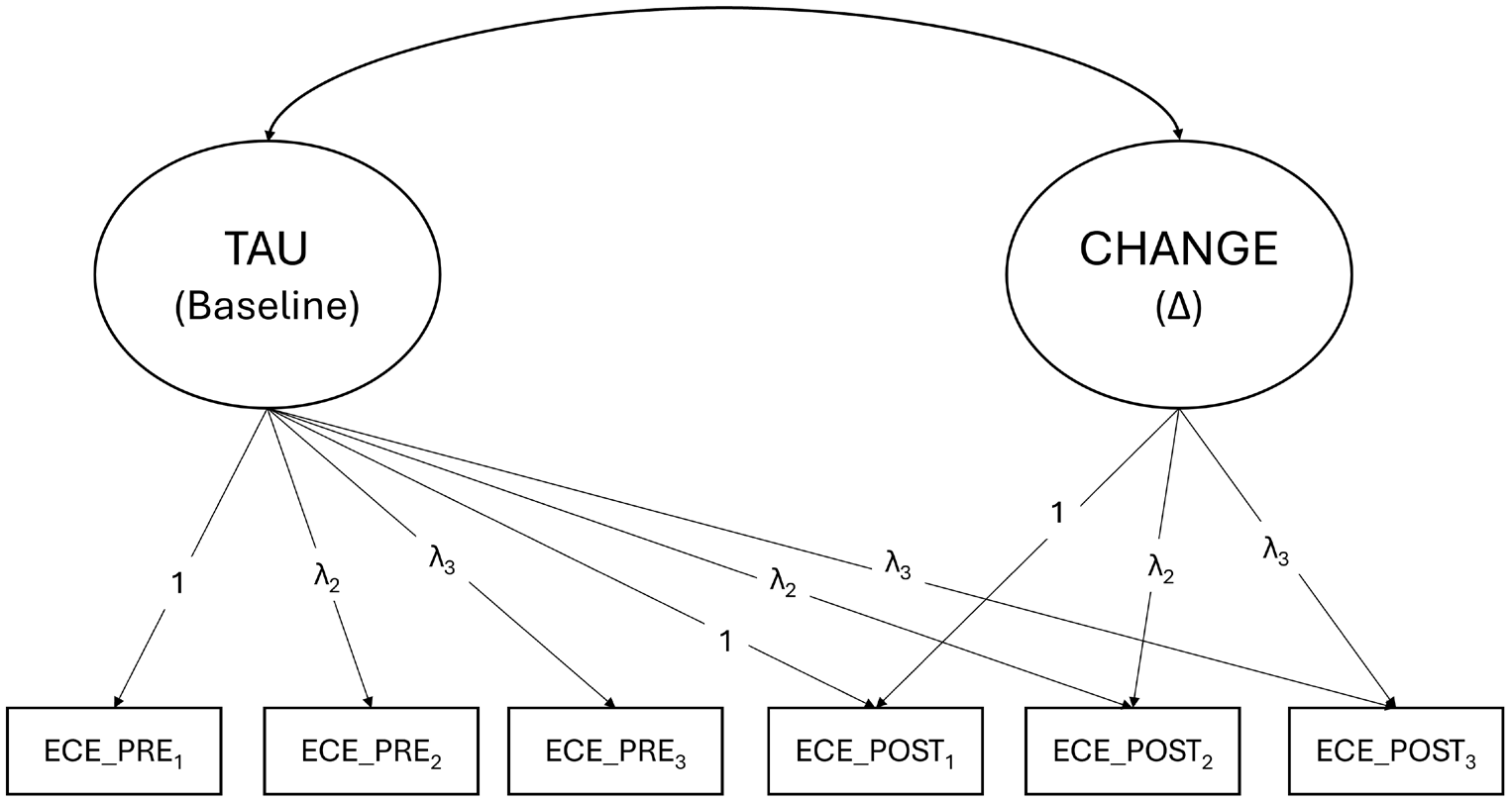
Conceptual representation of the reformulated (reduced form) latent change score model used in the present study. The model specifies two latent factors: TAU representing baseline ethnocultural empathy and CHANGE representing latent change in ethnocultural empathy between assessment occasions. Three indicators of ethnocultural empathy (ECE₁, ECE_2_, ECE₃) are measured at pre‐test and post‐test, with factor loadings (1, *λ*_2_, *λ*₃) constrained equal across time to ensure measurement invariance. Residual variances estimated for each indicator at each time point are omitted in the figure.

For each dimension, we used the corresponding three indicators measured at both time points. The measurement model was specified as follows: The TAU factor was defined by all six indicators (three pre‐test and three post‐test measures), with the first indicator's loading fixed at 1 for scale identification and the remaining loadings estimated but constrained to be equal across time points to ensure measurement invariance; the CHANGE factor was defined by the three post‐test indicators only, with factor loadings constrained to be identical to those of the TAU factor, ensuring that change was measured on the same scale as baseline levels; intercepts of the first indicator at both time points were fixed at zero for identification purposes, while the intercepts of the remaining indicators were freely estimated but constrained to be equal across time points to ensure measurement invariance. Item‐specific residuals were allowed to correlate across time points to account for indicator‐specific stability not captured by the latent factors. This specification allows for the direct estimation of: mean and variance of baseline levels (TAU), indicating average initial status and individual differences therein; mean and variance of change (CHANGE), indicating average game effect and individual differences in responsiveness; covariance between baseline levels and change, indicating whether game effects varied with initial status.

We first estimated an unconditional model without covariates for each dimension to examine overall gamified learning effects. Then, we estimated conditional models including gender, age and migratory background as covariates to assess whether demographic characteristics moderated the effects of the game experience.

Model fit was evaluated using multiple indices following established guidelines (Hu & Bentler, 1999; Kline, 2016). The chi‐square test was used as a preliminary fit indicator, with non‐significant values (*p* > .05) suggesting acceptable fit. However, given the chi‐square test's sensitivity to sample size, we primarily relied on additional indices: the root mean square error of approximation (RMSEA), with values ≤ .06 indicating good fit and values ≤ .08 indicating acceptable fit; the comparative fit index (CFI), with values ≥ .95 indicating good fit and values ≥ .90 indicating acceptable fit; and the standardized root mean square residual (SRMR), with values ≤ .08 considered acceptable.

In order to evaluate the robustness of the results to missing data assumptions, all models were re‐estimated using multiple imputation (MI) with 20 imputed datasets generated via Markov Chain Monte Carlo (MCMC) sampling. These were then analysed using the same model specifications as the main analysis. Parameter estimates were combined across imputed datasets using Rubin's rules.

## RESULTS

### Perceived educational scope of the game

Most participants reported improved empathy‐related skills after the JOINclusion gameplay experience. When asked whether the game helped them recognize and understand others' emotions, 61.5% of students responded ‘Yes’, 32.4% answered ‘Maybe’, and only 6.1% responded ‘No’. Similarly, 53.4% reported that JOINclusion helped them appreciate different perspectives and respond more empathetically, while 36.0% chose ‘Maybe’, and 10.7% selected ‘No’. Finally, 64.6% of participants indicated that the game increased their motivation to contribute to the well‐being of others, with 28.7% answering ‘Maybe’, and 6.7% responding ‘No’.

### Descriptive analyses

Item‐level descriptive statistics for the ethnocultural empathy scale are presented in Table 2. Given the non‐normal distribution of the items, indicated by significant Shapiro–Wilk test results, the subsequent LCS models were estimated using robust maximum likelihood (MLR) to account for non‐normality in the data.

**TABLE 2:** Means, Standard deviations and normality test statistics for pre‐ (*N* = 286) and post‐test scores (*N* = 172).

Item	Pre‐test mean (SD)			Post‐test mean (SD)			Cohen's *d*
	*M*	SD	Shapiro–Wilk statistic (*p*)	*M*	SD	Shapiro–Wilk statistic (*p*)	
EFE 1	2.64	.66	.58 (***)	2.65	.59	.62 (***)	.02
EFE 2	2.55	.65	.68 (***)	2.55	.63	.68 (***)	.00
EFE 3	2.56	.64	.67 (***)	2.60	.63	.65 (***)	.06
EPT 1	2.39	.72	.74 (***)	2.44	.61	.73 (***)	.08
EPT 2	2.21	.75	.79 (***)	2.30	.70	.78 (***)	.12
EPT 3	2.06	.76	.81 (***)	2.34	.70	.77 (***)	.38

*Note*: Cohen's *d* is interpreted as small (0.20), medium (0.50) or large (0.80).

*** *p* < .001.

### Latent change score models

The LCS model for the EFE dimension showed excellent fit with the data, SB*χ* ^2^ (12) = 11.891, *p* = .455; RMSEA = .000, 90% CI [.000, .060]; CFI = 1.000; SRMR = .039. All fit indices were within the acceptable ranges, suggesting that the model appropriately captured the underlying structure of the data.

The mean estimate for TAU was positive and statistically significant (2.508, *p* < .001), indicating that the baseline levels were significantly different from zero. The mean estimate for CHANGE was positive but only marginally significant (.061, *p* = .086, Cohen's d = .24), suggesting a small effect by conventional standards (Cohen, 1988) towards improvement in EFE following the game experience that approached conventional significance thresholds (*p* ≤ .05). Both variances for TAU and CHANGE were statistically significant (TAU variance = .08, *p* < .001; CHANGE variance = .07, *p* < .05), revealing significant individual differences in both baseline EFE levels and in participants' responsiveness to the game experience. The non‐significant covariance between TAU and CHANGE (*p* > .05) indicates that baseline EFE levels were not associated with the magnitude of change following the game experience.

When demographic covariates (i.e., gender, age, migratory background) were added to the model, none reached statistical significance (all *p*s > .05), suggesting that the effects of the gamified learning experience on EFE were consistent across these demographic characteristics.

The LCS model for the EPT dimension also demonstrated good fit with the data, SB*χ* ^2^ (12) = 21.351, *p* = .046; RMSEA = .052, 90% CI [.007–.088]; CFI = .948; SRMR = .057). The TAU score was significant (2.353, *p* < .001), indicating that baseline levels of EPT differed significantly from zero. The CHANGE coefficient was positive and statistically significant (.081, *p* = .019, Cohen's *d* = .27), representing a small but meaningful effect, thereby providing evidence of the game's effect on empathy perspective‐taking. Also, a significant negative correlation between change scores and baseline levels emerged (−.057, *p* = .002), indicating that participants who began with lower initial perspective‐taking abilities showed greater improvement following the game experience.

Similar to the EFE dimension, demographic covariates did not reach statistical significance in the EPT model (all *p*s > .05), suggesting that the positive effects of the gamified learning experience on perspective‐taking were consistent across gender, age and migratory background.

### Robustness check with multiple imputation

The analyses with MI suggested the robustness of the main results under the missing data assumptions. For the ECE dimension, the results were congruent with those from the main analysis. The mean of the stable trait factor TAU (MI: 2.515), the mean of the change factor (MI: 0.057, *p* = .081) and the TAU–Change covariance (MI: −0.017, *p* = .276) were consistent in both magnitude and significance pattern across the two approaches. Furthermore, the non‐significant change effect observed in the main analysis was replicated under MI, indicating that it does not reflect reduced statistical power due to missing data.

For the EFE dimension, the two approaches again converged on the same conclusions. The mean of TAU was almost identical (MI: 2.371), and the positive change factor mean was significant in both analyses (MI: 0.070, *p* = .039), as was the negative TAU–Change covariance (MI: −0.050, *p* = .007).

## DISCUSSION

The present study explored the efficacy of JOINclusion, a serious mobile game designed to foster ethnocultural empathy among primary school children from Greece, Italy and the Netherlands. The results indicate that the game shows promise as an educational tool: Participating children provided positive feedback on its perceived educational value, and quantitative analyses revealed pre‐post improvements in ethnocultural empathy, with notable gains in EPT.

### Perceived educational value

The prevalence of positive responses provided by children suggests that the gamified approach implemented in the JOINclusion game facilitated perceived improvements in ethnocultural competence. Many participants reported positive changes in their ability to empathize and recognize others' emotions.

Additionally, many participants felt they improved their ability to see things from others' perspectives. This finding is relevant, as it might be read as an indicator that the experiential learning provided by the game environment supported the cognitive decentring necessary for adopting alternative ethnocultural viewpoints (Davis, 1994; Todd & Galinsky, 2014).

Noteworthy, most children reported feeling more inclined to help others. This result is consistent with the empathy–altruism hypothesis (Batson et al., 2015), which posits that genuine empathic concern fosters prosocial motivation.

### Effects on empathy components

In the quantitative analysis of changes before and after the gameplay, the results revealed that the improvement in EPT was more pronounced than in EFE, where gains were marginal. Two factors likely explain this differential responsiveness, which reflect both developmental trajectories and intervention design characteristics.

From a developmental perspective, emotional empathy emerges earlier in childhood, while cognitive empathy matures later and may be more susceptible to external influences and structured interventions (Spinrad et al., 2022). Consistent with this, baseline EFE scores were already relatively high at pre‐test, whereas EPT scores started lower, thus leaving greater room for improvement. We interpret this pattern as consistent with Hoffman's (2000) distinction between emotional resonance and cognitive perspective‐taking.

From a design standpoint, JOINclusion's narrative architecture may be suited to engaging cognitive processes. In Story Mode, players are tasked with reflecting on social dilemmas and adopting alternative viewpoints. The Multiplayer Mode reinforces these cognitive gains by promoting cooperative problem‐solving, which requires real‐time perspective coordination with peers.

### Who benefits Most

A negative correlation emerged between baseline perspective‐taking scores and subsequent improvements, suggesting that children who started with lower scores benefited the most. This result may have both theoretical and practical implications. On the theoretical side, it suggests that JOINclusion may have acted as a scaffold for children who had not yet consolidated perspective‐taking skills through their developmental path. In practice, the game may have potential as an inclusive educational tool, helping to reduce within‐classroom disparities in social–emotional competence.

The analysis of demographic variables revealed no significant effect. That is, neither age, nor sex, nor migratory background was a factor that moderated the improvements that were observed. This suggests that the active mechanisms might operate through general developmental processes and not through specific social or cultural positions. However, the limitations listed below warrant caution with these conclusions.

### Practical implications

Beyond statistical effects, the observed gains may translate into meaningful behavioural shifts in children's daily experiences. These shifts may manifest in the form of providing support rather than ignoring, stepping in when others are excluded or starting cross‐group conversations. Such changes have the potential to significantly enhance the daily experiences of immigrant and minority children, who frequently experience exclusion. At the classroom level, these changes may lead to a shift towards more inclusive peer norms. It is important to note that middle childhood represents a sensitive period for both empathy consolidation and prejudice formation (Raabe & Beelmann, 2011). Thus, small gains during this developmental window can have a significant impact if they encourage openness to other groups.

Taken together, the findings offer preliminary insights that may inform the design of game‐based interventions aimed at promoting ethnocultural empathy in schools. One of JOINclusion's main strengths is its design, which is anchored in well‐established developmental and socio‐cognitive theories. This makes its core features transferable to other interventions.

Three main aspects might have contributed to the expected outcomes, though further research is needed to validate this study's findings and ensure their reliability. The first is progressive scaffolding through perspective‐reversal prompts (‘How would you feel?”), which draws on empathy‐induction methods. The second is narrative branching with visible consequences, which operationalizes vicarious learning. The third is contingent feedback that targets individual competency gaps, grounded in the principles of socio‐cognitive scaffolding. As each of these mechanisms is associated with a particular theoretical framework rather than with the JOINclusion game itself, they could be integrated into other serious games or classroom activities that are designed to achieve a similar objective.

The JOINclusion design may also offer advantages for scalability. Unlike traditional programs, which require extensive teacher training (Schonert‐Reichl et al., 2015), the narrator's contingent guidance reduces the reliance on teacher expertise, requiring only a brief orientation on session facilitation and post‐gameplay reflection.

Furthermore, JOINclusion's alignment with the CASEL (2020) framework positions it as a complementary tool within existing social–emotional learning programmes. The integration of a digital supplement in the form of a game could be a valuable addition to the SEL curricula implemented in educational institutions. This integration would serve to reinforce fundamental competencies, with a particular emphasis on enhancing social awareness and relationship skills.

At the policy level, JOINclusion provides a practical tool for addressing priorities related to intercultural competence development in the increasingly diverse educational contexts of Europe (European Commission, 2020). Education authorities might consider piloting the game across diverse school networks to assess effectiveness across contexts. This would precede a more widespread implementation. Teacher preparation programmes could also investigate their application as a pedagogical tool that demonstrates game‐based learning principles and serves as a content resource for modelling empathy instruction.

### Theoretical implications

Barrett's framework (2016, 2018) predicted that intercultural competence components—openness to ethnic and cultural differences and empathy—can be cultivated from primary school. Our data are consistent with that prediction. The results also point to Social and Emotional Learning as a promising foundation for developing ethnocultural empathy. SEL competences such as perspective‐taking, emotional awareness and prosocial behaviour are instrumental not only for classroom harmony but also for preparing students to engage constructively across cultural differences.

Overall, the findings of the current study support the idea that schools can function as transformative spaces for shaping how children perceive and respond to diversity. Structured exposure to intercultural situations, combined with opportunities to reflect on others' experiences and principles of fairness, can foster inclusive thinking and feeling that support social cohesion.

### Limitations

Despite the encouraging outcomes, several limitations of the present study must be acknowledged. First, the study assessed only immediate effects within a relatively condensed two‐week window, with participants completing the pre‐questionnaire and first session on the same day, followed shortly by the second session and post‐questionnaire. As a consequence, this timeframe did not allow the evaluation of the persistence and transfer of the observed changes to behaviour. It is possible that gains displayed immediately post‐intervention may not translate to sustained improvements in peer interactions, reduced bullying or intergroup relations.

Second, the absence of a control group represents a relevant constraint for causal inference, as observed changes may partly reflect maturation, testing effects or other unmeasured influences. Third, methodological constraints had a detrimental effect on statistical precision. Although our sample size aligns with typical published studies in this area, the statistical power remained insufficient for reliably detecting small effects across both empathy dimensions. Furthermore, the limited sample sizes within demographic and national subgroups, combined with moderate dropout rates, prevented comparative analyses. These power constraints also warrant caution when interpreting effect magnitudes. Although the observed effect sizes are in line with meta‐analytic benchmarks for school‐based SEL interventions (Durlak et al., 2011; Teding van Berkhout & Malouff, 2016), effect estimates from underpowered studies tend to be imprecise and potentially inflated (Button et al., 2013). Future research with larger, more balanced samples should examine potential moderating effects of gender, age and migratory background to identify whether certain student populations benefit differentially from game‐based empathy interventions.

Additionally, two measurement limitations should be noted. The questions assessing the perceived educational value of the game were developed ad hoc for this study and were not subjected to independent validation. Also, the ethnocultural empathy scale was reduced from its original form, which may have impacted construct coverage.

Furthermore, several potential moderators remain unexplored in the current study. Specifically, the study did not use engagement metrics, such as time‐on‐task or self‐reported immersion. The variability in engagement is hypothesized to have influenced outcomes, with students who displayed greater attentiveness and investment in the scenarios potentially benefiting more than those who responded in a more superficial manner. Also, the study did not examine whether structured post‐gameplay debriefing enhances transfer, how group composition influences Multiplayer outcomes, and whether these effects differ across classroom contexts. Similarly, the role of contextual moderators remains unexplored. Future research should examine contextual moderators that may shape the effectiveness of JOINclusion. In particular, studies should investigate how the quality of teacher facilitation, classroom composition, peer dynamics, school climate, family environments and broader cultural contexts influence children's engagement with the game and the resulting development of empathy. The incorporation of these multi‐level contextual factors into study designs will facilitate the clarification of the conditions under which the intervention is most beneficial, as well as the identification of the classroom, school and cultural environments that optimize its impact.

Finally, the current design does not permit disaggregating effects attributable to Story Mode from those attributable to Multiplayer Mode, as participants experienced both components sequentially. Future research could isolate the unique contribution of each modality and examine whether collaborative gameplay provides incremental benefit beyond individual practice.

## CONCLUSION

The findings of this study indicate that JOINclusion is a feasible approach for supporting ethnocultural empathy in middle childhood. The observed differential effects, with stronger gains in cognitive compared to affective empathy, align with developmental theory and the design logic of the game. Additionally, the compensatory pattern identified highlights particular value for children who require greater support in perspective‐taking.

Further refinement is necessary to achieve more substantial gains in emotional resonance and to ensure long‐term impact. Nevertheless, the observed improvements are promising. With continued development and rigorous evaluation, JOINclusion may contribute significantly to educational strategies that foster social inclusion in multicultural learning environments.

## AUTHOR CONTRIBUTIONS

**Alessandra Colella:** Conceptualization; data curation; investigation; writing – original draft. **Concetta Esposito:** Conceptualization; methodology; data curation; formal analysis; writing – original draft. **Annaleda Mazzucato:** Conceptualization; investigation; data curation; writing – original draft. **Ioannis Christidis:** Writing – review and editing. **Enrique Hortal:** Funding acquisition; project administration; writing – review and editing. **Helen C. Leligou:** Writing – review and editing. **Davide Marocco:** Conceptualization; methodology; writing – review and editing. **Michela Ponticorvo:** Conceptualization; methodology; writing – review and editing. **Yusuf Can Semerci:** Writing – review and editing. **Dario Bacchini:** Conceptualization; supervision; writing – review and editing.

## FUNDING INFORMATION

This project was carried out with the support of the Erasmus+ Programme of the European Union (Project Reference: 2021‐2‐NL01‐KA220‐SCH‐000048865; https://erasmus‐plus.ec.europa.eu/projects/search/details/2021‐2‐NL01‐KA220‐SCH‐000048865).

## CONFLICT OF INTEREST STATEMENT

The authors declare that they have no known conflicts of interest.

## ETHICS STATEMENT

The study was approved by the institutional review board of the University of Naples “Federico II.”

## INFORMED CONSENT

Informed consent was obtained from parents or legal guardians of the participants, while students provided their assent prior to participation.

## Supporting information


**Table S1.** Results from the confirmatory factor analysis (*N* = 286).

## Data Availability

The data that support the findings of this study are available from the corresponding author upon reasonable request.
