# Supplementary material for: JOINclusion: A serious mobile game for promoting ethnocultural empathy in schools
Source: Br J Dev Psychol. 2026 May 31;44(4):877–98. doi: 10.1111/bjdp.70055 (PMC13648312; doi:10.1111/bjdp.70055)
Supplement: Supplementary file 1 [file BJDP-44-877-s001.docx]

**Supplemental Material**

**JOINclusion: A serious mobile game for promoting ethnocultural empathy in schools**

**Supplemental Table**

**Table S1**

*Results from the Confirmatory Factor Analysis (N = 286)*

|  |  | Empathic Feeling and Expression (EFE) | Empathic Perspective Taking (EPT) |
| --- | --- | --- | --- |
| EFE 1 | I feel very sorry when I see someone being treated badly because of their ethnicity | .35*** |  |
| EFE 2 | If I see someone laughing at someone else's origins, I ask them to stop, even if it doesn't directly affect me | .54*** |  |
| EFE 3 | I like to help people of a different ethnicity than mine if I think someone is fooling them | .63*** |  |
| EPT 1 | I can imagine how some people might feel angry that they don't have the same opportunities because of the color of their skin or the country they're from |  | .56*** |
| EPT 2 | I can easily imagine how it feels to be a foreign child |  | .81*** |
| EPT 3 | It's easy for me to imagine myself in the life stories of people who come from countries other than mine |  | .55*** |

*Note*. Standardized coefficients are reported. *** *p* < .001. Covariance between the two factors: .38, *p* < .001.
